# Supplementary material for: Organic dry pea (Pisum sativum L.) biofortification for better human health
Source: PLoS One. 2022 Jan 13;17(1):e0261109. doi: 10.1371/journal.pone.0261109 (PMC8757916; doi:10.1371/journal.pone.0261109)
Supplement: Attachment — Submitted filename: Review-PONE-D-21-24900.pdf [file pone.0261109.s002.pdf]

PLOS ONE

Organic dry pea (Pisum sativum L.) biofortification for better human health

--Manuscript Draft--

**Manuscript Number:**	PONE-D-21-24900
**Article Type:**	Research Article
**Full Title:**	Organic dry pea (Pisum sativum L.) biofortification for better human health
**Short Title:**	Organic dry pea biofortification
**Corresponding Author:**	Dil Thavarajah<br>Clemson University<br>Clemson, SC UNITED STATES
**Keywords:**	Plant breeding, organic production, dry pea, biofortification, nutritional breeding, prebiotic carbohydrates, minerals
**Abstract:**	A primary criticism of organic agriculture is its lower yield and nutritional quality compared to conventional systems. Nutritionally, dry pea (Pisum sativum L.) is a rich source of low digestible carbohydrates, protein, and micronutrients. This study aimed to evaluate dry pea cultivars and advanced breeding lines using on-farm field selections to inform the development of biofortified organic cultivars with increased yield and nutritional quality. A total of 44 dry pea entries were grown in two USDA-certified organic on-farm locations in South Carolina (SC), USA, for two years. Seed yield and protein for dry pea ranged from 61 to 3833 kg/ha and 12.6 to 34.2 g/100 g, respectively, with low heritability estimates. Total prebiotic carbohydrate concentration ranged from 14.7 to 26.6 g/100 g. A 100-g serving of organic dry pea provides 73.5 to 133% of the recommended daily allowance (%RDA) of prebiotic carbohydrates. Heritability estimates for individual prebiotic carbohydrates ranged from 0.27 to 0.82. Organic dry peas are rich in minerals (Fe: 1.9-26.2 mg/100 g; Zn: 1.1-7.5 mg/100 g) and have low to moderate concentrations of phytic acid (18.8-516 mg/100 g). Significant cultivar, location, and year effects were evident for grain yield, thousand seed weight (TSW), and protein, but effects for other nutritional traits varied with genotype, environment, and interactions. “AAC Carver,” “Jetset,” and “Mystique” were the best-adapted cultivars with high yield, and “CDC Striker” had the highest protein concentration; these cultivars should be incorporated into organic dry pea breeding programs to develop cultivars suitable for organic production. In conclusion, organic dry pea has potential as a winter cash crop in southern climates but will require selecting diverse genetic material and location sourcing to develop improved cultivars with higher yield, disease resistance, and nutritional quality.
**Order of Authors:**	Dil Thavarajah
	Tristan J. Lawrence
	Sarah Powers
	Joshua Kay
	Pushparajah Thavarajah
	Emerson Shipe
	Rebecca McGee
	Shiv Kumar
	Rick Boyles
**Additional Information:**	
**Question**	**Response**
**Financial Disclosure**<br><br>Enter a financial disclosure statement that describes the sources of funding for the	The funders had no role in study design, data collection and analysis, decision to publish, or preparation of the manuscript.

Powered by Editorial Manager® and ProduXion Manager® from Aries Systems Corporation

work included in this submission. Review the submission guidelines for detailed requirements. View published research articles from *PLOS ONE* for specific examples.<br><br>This statement is required for submission and **will appear in the published article** if the submission is accepted. Please make sure it is accurate.<br><br>**Unfunded studies**<br>Enter: *The author(s) received no specific funding for this work.*<br><br>**Funded studies**<br>Enter a statement with the following details:<br>• Initials of the authors who received each award<br>• Grant numbers awarded to each author<br>• The full name of each funder<br>• URL of each funder website<br>• Did the sponsors or funders play any role in the study design, data collection and analysis, decision to publish, or preparation of the manuscript?<br>• **NO** - Include this sentence at the end of your statement: *The funders had no role in study design, data collection and analysis, decision to publish, or preparation of the manuscript.*<br>• **YES** - Specify the role(s) played.<br><br>* typeset	
**Competing Interests**<br><br>Use the instructions below to enter a competing interest statement for this submission. On behalf of all authors, disclose any competing interests that could be perceived to bias this work—acknowledging all financial support and any other relevant financial or non-financial competing interests.<br><br>This statement is required for submission and **will appear in the published article** if the submission is accepted. Please make sure it is accurate and that any funding sources listed in your Funding Information	The authors have declared that no competing interests exist.

later in the submission form are also declared in your Financial Disclosure statement.<br><br>View published research articles from *PLOS ONE* for specific examples.<br><br>**NO authors have competing interests**<br><br>Enter: *The authors have declared that no competing interests exist.*<br><br>**Authors with competing interests**<br><br>Enter competing interest details beginning with this statement:<br><br>*I have read the journal's policy and the authors of this manuscript have the following competing interests: [insert competing interests here]*<br><br>* typeset	
**Ethics Statement**<br><br>Enter an ethics statement for this submission. This statement is required if the study involved:<br><br>• Human participants<br>• Human specimens or tissue<br>• Vertebrate animals or cephalopods<br>• Vertebrate embryos or tissues<br>• Field research<br><br>Write "N/A" if the submission does not require an ethics statement.<br><br>General guidance is provided below. Consult the submission guidelines for detailed instructions. **Make sure that all information entered here is included in the Methods section of the manuscript.**	"N/A" if the submission does not require an ethics statement

**Format for specific study types**<br><br>**Human Subject Research (involving human participants and/or tissue)**<br>• Give the name of the institutional review board or ethics committee that approved the study<br>• Include the approval number and/or a statement indicating approval of this research<br>• Indicate the form of consent obtained (written/oral) or the reason that consent was not obtained (e.g. the data were analyzed anonymously)<br><br>**Animal Research (involving vertebrate animals, embryos or tissues)**<br>• Provide the name of the Institutional Animal Care and Use Committee (IACUC) or other relevant ethics board that reviewed the study protocol, and indicate whether they approved this research or granted a formal waiver of ethical approval<br>• Include an approval number if one was obtained<br>• If the study involved *non-human primates*, add *additional details* about animal welfare and steps taken to ameliorate suffering<br>• If anesthesia, euthanasia, or any kind of animal sacrifice is part of the study, include briefly which substances and/or methods were applied<br><br>**Field Research**<br><br>Include the following details if this study involves the collection of plant, animal, or other materials from a natural setting:<br>• Field permit number<br>• Name of the institution or relevant body that granted permission	
**Data Availability**<br><br>Authors are required to make all data underlying the findings described fully available, without restriction, and from the time of publication. PLOS allows rare exceptions to address legal and ethical concerns. See the PLOS Data Policy and FAQ for detailed information.	Yes - all data are fully available without restriction

A Data Availability Statement describing where the data can be found is required at submission. Your answers to this question constitute the Data Availability Statement and **will be published in the article**, if accepted.<br><br>**Important:** Stating 'data available on request from the author' is not sufficient. If your data are only available upon request, select 'No' for the first question and explain your exceptional situation in the text box.<br><br>Do the authors confirm that all data underlying the findings described in their manuscript are fully available without restriction?	
**Describe where the data may be found in full sentences. If you are copying our sample text, replace any instances of XXX with the appropriate details.**<br><br>• If the data are **held or will be held in a public repository**, include URLs, accession numbers or DOIs. If this information will only be available after acceptance, indicate this by ticking the box below. For example: *All XXX files are available from the XXX database (accession number(s) XXX, XXX.)*.<br>• If the data are all contained **within the manuscript and/or Supporting Information files**, enter the following: *All relevant data are within the manuscript and its Supporting Information files.*<br>• If neither of these applies but you are able to provide **details of access elsewhere**, with or without limitations, please do so. For example:<br><br>*Data cannot be shared publicly because of [XXX]. Data are available from the XXX Institutional Data Access / Ethics Committee (contact via XXX) for researchers who meet the criteria for access to confidential data.*<br><br>*The data underlying the results presented in the study are available from (include the name of the third party*	All relevant data are within the manuscript and its Supporting Information files.

and contact information or URL).<br>• This text is appropriate if the data are owned by a third party and authors do not have permission to share the data.<br><br>* typeset	
Additional data availability information:	Tick here if your circumstances are not covered by the questions above and you need the journal’s help to make your data available.

Powered by Editorial Manager® and ProduXion Manager® from Aries Systems Corporation

Manuscript Click here to access/download;Manuscript;Thavarajah et al 7-31-2021.docx

Organic dry pea (*Pisum sativum* L.) biofortification for better human health

Dil Thavarajah[1], Tristan J. Lawrence[1], Sarah E. Powers[1], Joshua Kay[1], Pushparajah Thavarajah[1], Emerson Shipe[1], Rebecca McGee[2], Shiv Kumar[3], Rick Boyles[3]

[1]Plant and Environmental Sciences, 113 Biosystems Research Complex, Clemson University, Clemson, SC 29634, USA.
[2]USDA Agriculture Research Service, Grain Legume Genetics and Physiology Research Unit, Washington State University, Pullman, WA 99164, USA.
[3]Biodiversity and Crop Improvement Program, International Centre for Agricultural Research in the Dry Areas (ICARDA), P.O. Box 6299, Rabat-Institute, Rabat, Morocco.
[4]Plant and Environmental Sciences, 210 Pitner Center, PeeDee Research and Education Center, 2200 Pocket Road, Florence, SC 29506, USA.

Author for correspondence: Dil Thavarajah, Tel: +18646562607, Email: dthavar@clemson.edu

Abstract: A primary criticism of organic agriculture is its lower yield and nutritional quality compared to conventional systems. Nutritionally, dry pea (*Pisum sativum* L.) is a rich source of low digestible carbohydrates, protein, and micronutrients. This study aimed to evaluate dry pea cultivars and advanced breeding lines using on-farm field selections to inform the development of biofortified organic cultivars with increased yield and nutritional quality. A total of 44 dry pea entries were grown in two USDA-certified organic on-farm locations in South Carolina (SC), USA, for two years. Seed yield and protein for dry pea ranged from 61 to 3833 kg/ha and 12.6 to 34.2 g/100 g, respectively, with low heritability estimates. Total prebiotic carbohydrate concentration ranged from 14.7 to 26.6 g/100 g. A 100-g serving of organic dry pea provides 73.5 to 133% of the recommended daily allowance (%RDA) of prebiotic carbohydrates. Heritability estimates for individual prebiotic carbohydrates ranged from 0.27 to 0.82. Organic dry peas are rich in minerals (Fe: 1.9-26.2 mg/100 g; Zn: 1.1-7.5 mg/100 g) and have low to moderate concentrations of phytic acid (18.8-516 mg/100 g). Significant cultivar, location, and year effects were evident for grain yield, thousand seed weight (TSW), and protein, but effects for other nutritional traits varied with genotype, environment, and interactions. "AAC Carver," "Jetset," and "Mystique" were the best-adapted cultivars with high yield, and "CDC Striker" had the highest protein concentration; these cultivars should be incorporated into organic dry pea breeding programs to develop cultivars suitable for organic production. In conclusion, organic dry pea has potential as a winter cash crop in southern climates but will require selecting diverse genetic material and location sourcing to develop improved cultivars with higher yield, disease resistance, and nutritional quality.

Keywords: Plant breeding, organic production, dry pea, biofortification, nutritional breeding, prebiotic carbohydrates, minerals

Introduction: Organic agriculture production has increased since the American Organic Foods Production Act of 1990. The USDA National Organic Standards Board describes organic agriculture as "*an ecological production management system that promotes and enhances biodiversity, biological cycles, and soil biological activity*" (USDA, 2016). Pulse crops, including dry pea (*Pisum sativum* L.), increase the ecological, economic, and social benefits of organic cropping systems via biological nitrogen (N) fixation, enhanced biodiversity, and creation of healthy food systems that can combat malnutrition and obesity. Organic agriculture is perceived as more environmentally friendly and sustainable than high-yielding conventional farming systems. Several studies support that notion, indicating organic farming systems provide a range of soil, biological, ecological, and other environmental benefits over conventional farming systems (Murphy *et. al*., 2007;Crowder and Reginold, 2015).

Dry pea is an excellent source of complex carbohydrates, protein, vitamins, and minerals (5,6). Dry peas are naturally rich in iron (Fe: 4.6-5.4 mg/100 g), zinc (Zn: 3.9-6.3 mg/100 g), and magnesium (Mg: 135-143 mg/100 g). In addition, dry pea is naturally low in phytic acid (PA) (4.9-7.1 mg/g of PA or 1.4-2 mg/g of phytic-P) despite very high total P concentrations (3.5-5 mg/g) (Amarakoon *et al.*, 2012, 2015; Ray *et al.*, 2014; Powers and Thavarajah, 2019; Powers *et al.*, 2020). Nutritionally, dry pea is a rich source of low digestible carbohydrates (12-15 g/100 g), protein (20-25 g/100 g), and essential amino acids (e.g., lysine and tryptophan) (Powers and Thavarajah, 2019; Powers *et al.*, 2020). Dry pea, in a symbiotic relationship with Rhizobium bacteria, can also fix atmospheric nitrogen , providing 75-120 kg of N per hectare for use by subsequent crops (Peoples *et al.*, 1995).

Consumer demand for pulses has increased due to the demand for plant-based protein (Ohr, 2020). However, organic farming systems face three significant global challenges: (1) maintaining crop productivity to produce enough food for a projected population of 9 billion in 2050, (2) delivering the expected nutritional quality as a human food and animal feed, and (3) maintaining ecological benefits, e.g., N and phosphorus (P) use efficiency (13). A primary criticism of organic agriculture is lower yield and nutritional quality compared to non-organic systems. Organic grains use soil nutrients derived from organic cover crop breakdown. Organic consumers believe organic foods are nutritionally superior and improve human health compared to conventional foods; however, organically grown grains typically have lower yields and nutritional quality than conventionally grown crops (Trewavas, 2001; Murphy *et al.*, 2007; Wiebe *et al.*, 2016). A meta-

analysis of over 10,000 organic farmers representing >800,000 hectares of organic farmland demonstrated that, averaged among food crops (wheat, maize, common bean, potato, and vegetables), the organic yield was 80% of conventional yield (Kniss *et al.*, 2016). The organic to conventional yield ratio varied with crop type, cultivars in production, and growing locations, highlighting the importance of regional breeding programs for organic production (Kniss *et al.*, 2016). Therefore, it is essential within the organic farming framework to focus on organic plant breeding, resulting in more suitable cultivars for organic production and delivering enhanced nutritional quality and nutrient bioavailability to combat micronutrient malnutrition, obesity, and overweight.

Current world pea production is 14.1 MMT on over 18 million acres, with US dry pea production representing about 7.1% of world production on 1,052,001 acres (FAO, 2020). The USDA does not report definite statistics on organic dry pea acreage. Still, the number of acres devoted to organic pulse crops is approximately 1.5-2% of total dry pea and lentil acreage. In 2011, certified organic dry peas and lentils were grown on more than 17,877 acres; North Dakota and Washington led with over 3,500 acres each (19). Yellow dry pea has become one of the popular cool-season legumes grown in SC during the winter. Carolina soils, especially in the Pee Dee region, have pH and soil phosphorus (P), potassium (K), and organic matter levels appropriate for dry pea germination, establishment, and growth. A rotational cropping system of dry pea and cereal has shown promise in sustainable, non-organic farming operations (20). Winter legumes provide weed control and available soil N and P for the following summer grain crop (Powers and Thavarajah, 2019). Developing crops for optimal performance in organic management systems requires integrating a range of traits, such as yield, agronomy, nutrient use efficiency, disease resistance, and nutritional quality. However, no breeding efforts have aimed to reduce the yield gap or increase the nutritional quality (i.e., biofortification) of dry pea for organic farming systems. Similarly, genomic and translational resources for selecting dry pea cultivars for organic production are also nonexistent.

With increasing societal nutritional needs for organically grown dry pea, biofortification brings organic plant breeding and nutritional sciences together to work on the persistent problems of human nutrition. In addition, biofortification of dry pea under organic systems will improve human nutrition, provide N and C benefits to subsequent cereal and vegetable crops, and increase nutrient use efficiency and biodiversity. Current organic pulse production depends on cultivars

that have been bred for non-organic production, but these are often not suited to organic production. For example, these cultivars may have a low grain yield, production issues (weed control, disease resistance, etc.), and low nutritional quality. The objectives of this study were to evaluate 44 dry pea entries in two on-farm locations for two years to determine grain yield and nutritional quality for human food, e.g., high protein, low digestible carbohydrates, and minerals as well as low phytate.

Materials and Methods

Materials**:** Standards, chemicals, and high-purity solvents used for prebiotic carbohydrate, minerals, and PA analysis were purchased from Sigma Aldrich Co. (St. Louis, MO), Fisher Scientific (Waltham, MA), VWR International (Radnor, PA), and Tokyo Chemical Industry (Portland, OR) and used without further purification. Water, distilled, and deionized (ddH_2O) to a resistance of ≥18.2 MΩ×cm (PURELAB flex 2 system, ELGA LabWater North America, Woodridge, IL) was used for sample and reagent preparation.

Field design**:** The experimental field design was a randomized complete block design (RCDB) with 44 dry pea entries (25 cultivars and 19 advanced breeding lines) with two replications at two locations in 2019 and three replications at one location in 2020 (n=308; **Table 1**). The commercial dry pea cultivars were purchased from Pulse USA (Bismark, ND, USA), Meridian Seeds (Mapleton, ND, USA), and the Washington State Crop Improvement Association (Pullman, WA, USA). The advanced dry pea breeding accessions were obtained from the USDA-ARS Pulse Breeding Program, Washington State University, WA, USA **(Table 1)**. Material transfer agreements (MTAs) were signed with the seed companies and the USDA-ARS for field testing these entries in SC, USA. These dry pea cultivars were selected based on yield potential, disease resistance, and consumer acceptability. Before sowing, two soil samples were randomly taken at 0-6″ depth from each plot. The soil samples were homogenized, and three composite samples were analyzed for soil properties at the Clemson University Soil Testing laboratory, SC, USA. Soil properties, precipitation, and temperature varied with growing location **(Tables 2 and 3)**.

Land preparation: USDA-certified organic on-farm locations were WP Rawl and Sons (Pelion, SC, USA) and Calhoun Fields Laboratory (Clemson University, SC, USA). Before planting, fields were tilled using a disc harrow and smoothly leveled. All plots were then marked with a weatherproof barcoded field tag, and cultivar "Hampton" was planted as a control to eliminate the border effect. A cone plot planter was used for sowing seed in 1.4×6 m plots (8.4 m^2) containing

seven rows spaced 20 cm (7.9 in) apart, with a seeding depth of 5-7 cm (~2-3 in), at a seeding rate of 90 seeds/m^2. USDA-certified organic inoculant (Peaceful Valley Farm Supply, Inc, USA) was added to the seed packets at the rate of 3.1 g per kg of seed. Organically certified fertilizers, pesticides, and chemicals were not used in this experiment; weeds were removed by a mechanical cultivator attached to a small tractor. Irrigation was not provided. These cultivars and breeding lines were planted in mid-January and harvested in the third week of May. At physiological maturity (110-115 days after planting), the plots were harvested using a small plot. Dry pea grain yield was calculated based on the size of the plot, and 1000-seed weight (TSW) was calculated from the weight of 100 seeds, measured using a top-loading electronic balance. Subsamples (500-750 g) of harvested seeds were stored at −10 °C until nutritional quality analysis. Additional dry pea samples collected from each replication were hand cleaned, finely ground using a UDY grinder, and then stored at −10 °C until nutritional quality analysis. All nutritional quality data are reported on a dry basis (15% moisture).

Protein analysis: Finely ground dry pea samples were sent to the Soil Testing Laboratory, Clemson University, SC, for total N analysis, and then values converted to total protein content by multiplying by 6.25.

Prebiotic carbohydrate analysis: Dry pea seeds were ground (Blade Coffee Grinder, KitchenAid, St. Joseph, MI, USA) and sieved to 0.5-mm particle size. Carbohydrates were extracted the method described by Muir *et al.*, 2009. Ground dry pea samples (150 mg) were weighed into a centrifugal polypropylene tube (VWR International, Radnor, PA, USA). After adding 10 mL of water, each tube was mixed on a vortex mixer and placed in a water bath for 1 h at 80 ℃. Tubes were then centrifuged at 3000 g for 10 min, and the supernatant was filtered through a 13 mm × 0.45 μm nylon syringe filter (Thermo Fisher Scientific, MA, USA) into an HPLC vial. Carbohydrate analysis was done using a Dionex ICS-5000+ system (Thermo Scientific, Waltham, MA, USA) equipped with a pulsed amperometric detector (PAD) with a working gold electrode and a silver-silver chloride reference electrode. Analyte separation was achieved using a Dionex CarboPac PA1 analytical column (250 × 4 mm) in series with a Dionex CarboPac PA1 guard column (50 × 4 mm). Pure standards were used to identify peaks, generate calibration curves, and monitor detector sensitivity; a lab reference sample was also used to monitor extraction consistency. Concentrations were quantified within a linear range of 0.1-500 ppm with a minimum detection limit of 0.1 ppm. Concentrations of each carbohydrate were calculated according to $X = (C \times V) /$

m, where X is the moisture-corrected analyte concentration in the sample, C is the concentration in the filtrate, V is the sample volume, and m is the mass of the sample.

Starch analysis: Resistant, non-resistant, and total starch were measured using the modified Megazyme resistant starch assay method (22). Samples (100 mg) of finely ground seed were weighed into centrifugal polypropylene tubes, to which an enzyme solution (2 mL) containing amyloglucosidase (3 U/mL) and α-amylase (10 mg/mL) in sodium maleate buffer (100 mM, pH 6.0) was added. Tubes were then incubated with constant circular shaking (200 strokes/min) for 16 h at 37 ℃. Ethanol (4 mL; 99%) was added, then the tubes were vortexed, centrifuged at 1500 g for 10 min, and decanted into 100-mL volumetric flasks. Two additional washings were performed by adding 2 mL of ethanol (50%) and vortex mixing to suspend the pellet, followed by an additional 6 mL of ethanol (50%), vortex mixing, centrifugation, and decanting. Pooled non-resistant starch washings were brought to 100 mL volume with water. Pellets containing resistant starch were dissolved in 2 mL of 2 M KOH with a magnetic stir bar for 20 min in an ice water bath. Sodium acetate buffer (8 mL, 1.2 M, pH 3.8) was added, immediately followed by 0.1 mL of amyloglucosidase (AMG; 3300 U/mL). Samples were incubated at 50 ℃ in a water bath for 30 min. Tubes were then centrifuged (1500 g for 10 min). Resistant starch (RS) and non-resistant starch fractions were quantified via spectrophotometry. Starch solution (0.1 mL) and glucose oxidase/peroxidase (GOPOD) reagent (3 mL) were added to glass tubes and incubated for 20 min at 50 ℃. A glucose standard (1 mg/mL in 0.2% benzoic acid) was included in each batch. Absorbance was measured at 510 nm against a reagent blank. Non-resistant starch (NRS) was calculated using the formula NRS (g/100 g sample) = $\Delta E \times F/W \times 90$, where ΔE is the absorbance of the sample, F is the absorbance to microgram conversion factor (100 / absorbance of glucose standard), W is the sample dry weight, and 90 includes adjustments for volume, unit conversions, and free to anhydrous glucose. A similar formula was used to calculate resistant starch (RS), RS (g/100 g sample) = $\Delta E \times F/W \times 9.27$, where 9.27 includes adjustments for volume, unit conversions, and free to anhydrous glucose. Total starch (TS) was calculated as TS = RS + NRS.

Statistical analysis*:* Replicates, years and genotypes were included as class variables. Data from both years were combined (after testing for heterogeneity) and analyzed using a general linear model procedure (PROC GLM) mixed model (SAS Institute 9.4, 2012). Fisher's least significant difference (LSD) at ≤ 0.05 was performed for mean separation. Correlations (Pearson correlation coefficients) among yield, TSW, and other traits were determined. ANOVA was used to determine

if the effect was significant. A statistical model was developed to estimate broad-sense heritability (H^2) with the variables and genotype as random effects. The model was calculated using the restricted maximum likelihood (REML) method. H^2 was estimated as the proportion of variance due to genotype, and analyses were performed using JMP 14.0.0 and SAS 9.4.

Results

Field weather and soil conditions: The field trials took place at Clemson and Pelion, SC during 2019 and at Pelion, SC in 2020. A total of 25 cultivars and 19 breeding lines were evaluated at each location, with two replicates in 2019 due to seed limitations and three replicates in 2020 (n = 308) **(Table 1)**. In 2019, the Pelion, SC location was warmer (25.6 °C) and received more precipitation (68.6 mm) in May than the Clemson, SC location. In 2020, the average temperature was lower (20.8 °C) and the average precipitation was higher (236 mm) at Pelion, SC than in the previous year **(Table 2)**. In 2019, the Clemson field had a lower pH (6.3), with higher N-NO_3 (48 ppm), K (284 lbs/ac), and organic matter (4.3%) than the Pelion field, which had more P (727 lbs/ac). In 2020, Pelion soil values reflected higher pH (6.8 to 7.1), N-NO_3 (16 to 21 ppm), and organic matter (0.8 to 1.1%) compared to 2019 as well as lower levels of P (727 to 549 lbs/ac) and K (108 to 81 lbs/ac) **(Table 3)**. Clemson soils are clay loam, and Pelion soils are sandy, which may explain the differences in N, K, and organic matter.

Analysis of variance: With respect to yield, cultivar, year, and cultivar × location were highly significant at $P<0.05$, location and cultivar × year were significant at $P<0.1$, and all components were highly significant ($P<0.05$) for TSW **(Table 4)**. Only cultivar × location was not significant for protein, with all other components highly significant ($P<0.05$) **(Table 4)**. Broad-sense heritability estimates indicated TSW was more heritable (H^2=0.69) than yield (H^2=0.21) and protein (H^2=0.24). Most prebiotic carbohydrates varied with dry pea cultivar except for maltose and starch polysaccharides. For sugar alcohols, location was not significant for xylitol and mannitol, year was not significant for sorbitol, cultivar × location was not significant for mannitol, and cultivar × year was not significant for sorbitol; all other components were significant ($P<0.05$) for each sugar alcohol **(Table 4)**. For simple sugars, only cultivar and location significantly ($P<0.05$) affected glucose concentration, and only location and year were significant ($P<0.05$) for maltose concentration. Cultivar × location was not significant for fructose concentration, and cultivar × year was not significant for sucrose concentration. Location was not significant for arabinose concentration, with all other components being highly significant ($P<0.05$) for simple

sugars. For RFO and FOS, location was not significant for Ver+Kes, and cultivar × location was not significant for nystose, with all other components significant (P<0.1 and P<0.05) for each RFO and FOS **(Table 4)**. Location (P<0.05), year (P<0.1), and cultivar × year (P<0.05) had significant effects on resistant starch, while only location and year were significant (P<0.05) for total starch. Prebiotic carbohydrates exhibited broad ranges of heritability for organic dry pea, with glucose and fructose having the lowest heritability at 0.29 and 0.27, respectively. Galactinol (H^2=0.74) and Ver+Kes (H^2=0.75) had the highest heritability, with all other prebiotic carbohydrates having moderate to high heritability, except for maltose and the starch polysaccharides, which were not heritable. For mineral concentrations, cultivar was significant for all minerals except Se; cultivar × location was only significant for K (P<0.1) and Fe (P<0.05), and cultivar × year was not significant for any mineral **(Table 4)**. Location was significant (P<0.05) for K, Ca, Mg, Fe, Zn, and Se but not for P, Mn, and Cu. Additionally, the year was significant (P<0.05) for K, Ca, Fe, Zn, and Se but not for Mg, P, Mn, and Cu. Finally, only cultivar (P<0.1) and year (P<0.05) were significant for PA concentration of organically grown dry pea **(Table 4)**. All minerals were found to be not heritable.

Nutritional quality: Organic dry pea shows broad phenotypic variation for protein (12.6-34.2 g/100 g), prebiotic carbohydrates (12.5-19.8 g/100 g), minerals, and PA (88.8-354 mg/100 g) **(Table 5)**. Organic dry pea can provide a significant portion of the recommended daily allowance (RDA) of prebiotic carbohydrates (81%), protein (38-46%), and a range of minerals **(Table 5)**. Organic dry pea provides a significant amount of the %RDA for K (29.6-38.8%), Mg (31.3-40.3%), Zn (29.1-40%), and Se (36.4%) for both men and women but is not a good source of Ca (7.8-9.4%) in the diet **(Table 5)**.

Cultivar responses: Yield varied among the organically grown cultivars, with "AAC Carver" having the highest yield (~2600 kg/ha) and "LG Koda" the lowest (~750 kg/ha) **(Figure 2)**. "AAC Carver" had one of the lowest protein concentrations (~19 g/100g), while "CDC Striker," which had one of the lowest yields (~1000 kg/ha), had the highest protein concentration (~24 g/100 g) **(Figure 2)**. Cultivars varied in terms of the total concentrations of the sugar alcohols myo-inositol, xylitol, galactitol, sorbitol, and mannitol **(Figure 3A)**. The cultivar "Hampton" had the lowest concentration of sugar alcohols (~425 mg/100 g) and "CDC Greenwater" the highest (575 mg/100 g) **(Figure 3A)**. All cultivars had varying concentrations of RFOs (Raf+Sta and Ver+Kes), with

cultivar "Fiddle" having the lowest total RFO concentration (~5200 mg/100 g) and cultivar "Mystique" the highest (~6000 mg/100 g) **(Figure 3B)**.

Analysis using Pearson's correlation was performed to determine significant correlations between agronomic and nutritional quality traits **(Figure 1)**. A significant ($P<0.05$) and strong correlation was observed for total water-soluble carbohydrates and yield (r=0.42), with low but significant ($P<0.05$) positive correlations found between TSW and yield (r=0.2), and TSW and total water-soluble carbohydrates (r=0.26) **(Figure 1)**. Protein was significantly ($P<0.05$) negatively correlated with all agronomic traits: yield (r=−0.2), TSW (r=−0.26), and total water-soluble carbohydrates (r=−0.1) **(Figure 1)**. More specifically, significant ($P<0.05$) negative correlations were found between yield and xylitol, mannitol, sucrose, arabinose, maltose, and resistant starch, but the yield was significantly ($P<0.05$) positively correlated with galactinol, sorbitol, glucose, fructose ($P<0.1$), all RFO and FOS, as well as soluble starch and total starch **(Table 6)**. Finally, yield was not correlated with Zn, P, or PA but was positively correlated with both Mg ($P<0.05$) and Fe ($P<0.1$). A significant ($P<0.1$) negative correlation was observed between yield and K **(Table 7)**. Positive, significant correlations were evident for protein and myo-inositol ($P<0.1$), xylitol ($P<0.1$), mannitol ($P<0.05$), sucrose ($P<0.1$), arabinose ($P<0.05$), and maltose ($P<0.05$). Protein was predominantly negatively correlated with RFO and FOS carbohydrates ($P<0.05$) **(Table 6)**. All minerals were significantly ($P<0.05$) positively correlated with each other, while PA was negatively correlated with all minerals, especially Zn ($P<0.05$) **(Table 7)**.

Discussion: Organic pulse crop production is challenging for many reasons, one being the less suitable cultivars adapted for low-input organic systems. Current dry pea cultivars in North America are mainly bred for conventional production systems that use chemical herbicides and pesticides for weed, pest, and disease management. This paper reports the first detailed field study conducted in USDA Organic Certified fields to assess the performance of dry pea cultivars and advanced breeding lines under organic field conditions without adding any chemical fertilizers or herbicides. Our study clearly indicates "AAC Carver," "Jetset," and "Mystique" are the highest yielding dry pea cultivars (above 2000 kg/ha) and are the most suitable for organic production without a yield penalty **(Figure 1)**. The average crude protein content of the cultivars studied is ~21.1 g/100 g, with "CDC Striker" being the highest and "AAC Carver" the lowest **(Figure 1)**. Our on-farm organic field trials provide a thorough evaluation of available dry pea cultivars for

yield, protein, and other nutritional traits for two years. The information from this study will help organic producers decide if these dry pea cultivars will be profitable on their farm and, if so, which cultivar will perform best in their organic cropping system in terms of yield and protein. In addition, these data are very useful for future organic dry pea cultivar development with respect to selecting appropriate parents for organic systems.

Weed management in organic systems is a significant challenge. Dry pea is not a good weed competitor. Yield losses in organic systems can be up to 80% due to post-emergent weeds in the Northern Great Plains of Canada (Leeson *et al.*, 2000; Baird *et al.*, 2009; Shirtliffe and Johnson, 2012). Suggested methods to reduce weed pressure in an organic cropping system are to increase seeding rate, crop rotation, and seeding depth and to change planting dates. In Canada, dry pea reached a maximum economic return at a seeding rate of 200 seed/m^2 with a grain yield of 1725 kg ha^{-1}(Baird *et al.*, 2009). We used dry pea as a winter crop (Jan-May) in SC with 90 seeds/m^2 and manually reduced the post-emergence weeds, and several dry pea cultivars tested (7 out of 25) reached more than the threshold yields reported by the Canadian study **(Figure 1)**. Additionally, organic dry pea grain yields in the present study significantly varied with cultivar, year, and the interaction of cultivar × location ($P<0.05$), indicating cultivar performance is subject to growing conditions, e.g., soil, weather, and organic management conditions. Overall, average dry pea grain yield (769-2638 kg ha^{-1}) and protein concentrations (19.3- 24.2 mg/100 g) from this study are similar to results reported for studies in Canada and Australia (Baird *et al.*, 2009; Gollner *et al.*, 2019).

Pulse crops show great potential for biofortification and are suitable for meeting increasing consumer demand for organic plant-based protein, prebiotic carbohydrates, and minerals, especially within allergen- and gluten-free markets (Ray *et al.*, 2014; Johnson *et al.*, 2013; Thavarajah *et al.*, 2017). Our results indicate organic dry peas are rich in prebiotic carbohydrates (12.5-19.8 g/100 g), providing 63-99% of the RDA for adults **(Table 5)**. Sugar alcohols and RFOs have moderate to high broad-sense heritability (0.42-0.75) estimates, indicating it is possible to breed for variable concentrations of these prebiotic carbohydrates for better human health. Sucrose and arabinose are heritable traits, but starch polysaccharides are not **(Table 4)**. Total water-soluble carbohydrates (carbohydrates without starch polysaccharides) are significantly and positively correlated with grain yield and TSW but negatively correlated with seed protein content **(Figure 2)**. Organic dry pea prebiotic carbohydrate concentrations reported in this study are similar the

values reported in the literature (Wang *et al.*, 2009, 2011; Johnson *et al.*, 2013, 2015; Vandemark *et al.*, 2020). Prebiotic carbohydrates are critical components in healthy diets, supporting healthful hindgut microflora. Healthy gut microbiota decrease host obesity, inflammatory bowel diseases, and colorectal cancers and modulate immunological functions by affecting the growth and functioning of host cells (Ley et al., 2005). Due to the dietary nature of human metabolic disorders related to obesity, solutions will necessarily have a focus on a diet – *i.e.*, a cup of pulses a day provides 13-15 g of prebiotic carbohydrates and a range of micronutrients (Amarakoon *et al.*, 2012; Powers and Thavarajah, 2019; Powers *et al.*, 2020). Changing the levels of these prebiotic carbohydrates is possible by developing molecular markers for marker-assisted breeding with conventional breeding methods in pulse crops; however, genome-wide association mapping studies with diverse populations at several field locations are essential to avoid the yield and protein penalty by changing certain carbohydrates as a result of the quantitative nature of these nutritional traits (Johnson et al., 2020; 2021).

Pulses crops, including dry pea, also known as "poor man's meat," are low in fat and provide significant quantities of dietary protein (20-25 g/100 g) and minerals (Ray *et al.*, 2014; Thavarajah *et al.*, 2015). A 50-g serving of conventional grown dry pea provides 3.7-4.5 mg of Fe, 2.2-2.7 mg of Zn, and 22-34 µg of Se and is very low in PA (2.5-4.4 mg g^{-1}), which decreases the bioavailability of minerals (5,6). Similar to previous studies, our results show organic dry peas are also rich in Fe, Zn, and Se, but not a good source of Ca **(Table 5)**. Integrating genome-wide research approaches with conventional plant breeding to identify genetic markers associated with these mineral traits could significantly accelerate biofortification efforts by enabling molecular screening of exotic germplasm collections and elite cultivars (Johnson *et al.*, 2021; Powers *et al.*, 2021).

A rotational cropping system of dry peas and cereals has been promised in organic and non-organic farming systems (Olesen *et al.*, 2000; Gan *et al.*, 2015). Dry pea as a winter cash crop will provide economic and environmental benefits of weed control and soil nutrient management for smallholder organic farms. Generally, organic producers use legume or grass-legume mix cover crops for their winter season to increase soil fertility and weed control (Snapp *et. al.*, 2005; Thavarajah *et al.*, 2019;). Overall, critical issues for organic pulse crop production are (1) production system issues: breeding and selection of high yielding varieties adapted for organic cropping systems and growing regions; (2) nutritional quality and grading: organic edible pulse

markets are susceptible to nutritional quality, so it is difficult to sell anything less than top-grade, i.e., high protein; (3) marketing and trade: for example, the organic grain market remains small as a result of a limited number of buyers in a given region; and (4) public research availability: minimal research is available on organic pulse production, variety development, nutritional quality, and end-use as a whole food or an ingredient (Trewavas, 2001; Kniss *et al.*, 2016). Moreover, no research has been conducted regarding reducing the yield gap without compromising nutritional yield and developing genomic tools for marker-assisted breeding of organic pulse cultivars, i.e., biofortification of organic pulse grains. Therefore, it is essential within the organic farming framework to focus on organic plant breeding activities that will result in cultivars that are more suitable for organic production environments and will deliver economic and social benefits to growers and consumers. Overall, organic markets (especially the gluten-free market) will continue to grow >10-20% per annum at the retail sales level for the foreseeable future in all food categories due to increasing awareness of the connection between diet and human health (Ohr, 2020). Successful production of organic pulse crops would increase regional production acreage, grower profitability, and stakeholder confidence in organic farming systems in the USA.

Conclusions: Organic dry pea is a potential winter crop in southern US regions. Dry pea grain yields and protein concentrations are within the range of conventional production systems. Further, organic dry pea is a rich source of prebiotic carbohydrates (14.7-26.6 g/100 g). Most individual prebiotic carbohydrates are moderate to high in terms of broad-sense heritability estimates, with the exception of starch polysaccharides. Organic dry peas are rich in minerals with low to moderate concentrations of phytic acid. “AAC Carver,” “Jetset,” and “Mystique” demonstrated the highest yields and “CDC Striker” the highest protein concentrationt. These cultivars can be incorporated into organic dry pea breeding programs to develop cultivars suitable for organic production. Finally, organic dry pea production has potential as a winter cash crop in southern climates; this can be accomplished by selecting diverse genetic material and location sourcing to develop improved cultivars with a higher yield, disease resistance, and nutritional quality. On-farm evaluation of dry pea cultivars and advanced breeding lines under organic management provides valuable information for growers, allowing them to make critical decisions regarding variety selection for (1) growing location, (2) organic management practice, and (3) intended end-use or

nutritional quality (prebiotic carbohydrates, protein, minerals, and low phytate), all of which are critical for maximizing grower productivity, profitability, and socio-economic status.

Acknowledgments: Funding support for this project was provided by the Organic Agriculture Research and Extension Initiative (OREI) (award no. 2018-51300-28431/proposal no. 2018-02799) of the United States Department of Agriculture, National Institute of Food and Agriculture, and the USDA-ARS Pulse Health Initiative (DT and RB); the Specialty Crop Block Grant Program of the U.S. Department of Agriculture through grant AM180100XXXXG026 (DT); the USDA National Institute of Food and Agriculture, [Hatch] project [1022664] (DT); and the Good Food Institute (DT). Its contents are solely the responsibility of the authors and do not necessarily represent the official views of the USDA. We thank Bradley Stancil (Crop Improvement, Clemson University), David Robb (Organic Farm, Clemson University), Sorghum Breeding and Genetics (Drs. Boyles and Kresovich team), and Charles Wingard and Ben Dubard from WP Rawl & Sons, Inc., for field operations; and Martin Hochhalter (Meridian Seeds), Tyler Kres (Pulse USA), the Washington State Crop Improvement Association, and the USDA-ARS for providing dry pea seeds.

Amarakoon D, Thavarajah D, McPhee K, Thavarajah P. Iron-, zinc-, and magnesium-rich field peas (Pisum sativum L.) with naturally low phytic acid: A potential food-based solution to global micronutrient malnutrition. J Food Compos Anal. 2012;27(1).

Amarakoon D, Thavarajah D, Sen Gupta D, McPhee K, DeSutter T, Thavarajah P. Genetic and environmental variation of seed iron and food matrix factors of North-Dakota-grown field peas (Pisum sativum L.). J Food Compos Anal. 2015;37.

Baird JM, Shirtliffe SJ, Walley FL. Optimal seeding rate for organic production of lentil in the northern Great Plains. Can J Plant Sci. 2009 Nov 1;89(6):1089–97. Available from: https://doi.org/10.4141/CJPS08226.

Clark A. Managing cover crops profitably [Internet]. Third Edit. Diane Publishing; 2008. 135–141 p. Available from: https://books.google.com/books?hl=en&lr=&id=ahxLEpn6WYwC&oi=fnd&pg=PP2&dq=11.%09Clark,+A.+2008.+Field+Peas.+Managing+Cover+Crops+Profitably,+Third+Edition.+Sustainable+Agriculture+Research+and+Education+Program,+135-141.+Print&ots=aKuJg-wDTx&sig=_QVYFiOgA.

Crowder DW, Reganold JP. Financial competitiveness of organic agriculture on a global scale. Proc Natl Acad Sci [Internet]. 2015 Jun 16 [cited 2020 Jan 7];112(24):7611–6. Available from: https://www.pnas.org/content/112/24/7611.

FAO. FAOSTAT [Internet]. FAOSTATS- Crops. 2020 [cited 2020 Dec 9]. Available from: http://www.fao.org/faostat/en/#data/QC.

Gan Y, Hamel C, O'Donovan JT, Cutforth H, Zentner RP, Campbell CA, et al. Diversifying crop rotations with pulses enhances system productivity. Sci Rep. 2015;5(1):14625. Available from: https://doi.org/10.1038/srep14625.

Godfray HCJ, Beddington JR, Crute IR, Haddad L, Lawrence D, Muir JF, et al. Food Security: The Challenge of Feeding 9 Billion People. Science. 2010 Feb 12;327(5967):812 LP – 818. Available from: http://science.sciencemag.org/content/327/5967/812.abstract.

Gollner G, Starz W, Friedel JK. Crop performance, biological N fixation and pre-crop effect of pea ideotypes in an organic farming system. Nutr Cycl Agroecosystems. 2019;115(3):391–405. Available from: https://doi.org/10.1007/s10705-019-10021-4.

Institute of Medicine of the National Academies. Dietary Reference Intakes for Energy, Carbohydrate, Fiber, Fat, Fatty Acids, Cholesterol, Protein, and Amino Acids (Macronutrients). National Academies Press: Washington, D.C., 2005.

Johnson CR, Thavarajah D, Combs GF, Thavarajah P. Lentil (Lens culinaris L.): A prebiotic-rich whole food legume. Food Res Int. 2013;51(1).

Johnson CR, Thavarajah D, Thavarajah P, Fenlason A, McGee R, Kumar S, et al. A global survey of low-molecular weight carbohydrates in lentils. J Food Compos Anal. 2015;44.

Johnson N, Boatwright JL, Bridges W, Thavarajah P, Kumar S, Shipe E, et al. Genome-wide association mapping of lentil (Lens culinaris Medikus) prebiotic carbohydrates toward improved human health and crop stress tolerance. Sci Rep. 2021;11(1):13926. Available from:

https://doi.org/10.1038/s41598-021-93475-3.

Johnson N, Johnson CR, Thavarajah P, Kumar S, Thavarajah D. The roles and potential of lentil prebiotic carbohydrates in human and plant health. PLANTS, PEOPLE, PLANET. 2020 Mar 22. Available from: https://doi.org/10.1002/ppp3.10103.

Kniss AR, Savage SD, Jabbour R. Commercial crop yields reveal strengths and weaknesses for organic agriculture in the United States. PLoS One. 2016 Aug 1;11(8).

Kniss AR, Savage SD, Jabbour R. Correction: Commercial Crop Yields Reveal Strengths and Weaknesses for Organic Agriculture in the United States. PLoS One. 2016 Nov 8;11(11):e0165851. Available from: https://doi.org/10.1371/journal.pone.0165851.

Leeson JY, Sheard JW, Thomas AG. Weed communities associated with arable Saskatchewan farm management systems. Can J Plant Sci. 2000 Jan 1;80(1):177–85. Available from: https://doi.org/10.4141/P99-039.

Ley RE, Bäckhed F, Turnbaugh P, Lozupone CA, Knight RD, Gordon JI. Obesity alters gut microbial ecology. Proc Natl Acad Sci U S A [Internet]. 2005 Aug 2;102(31):11070 LP – 11075. Available from: http://www.pnas.org/content/102/31/11070.abstract.

Linda Milo Ohr. Plant-Based Protein Market Grows Stronger. Food Technology Magazine. 2020;74(10). Available from: https://www.ift.org/news-and-publications/food-technology-magazine/issues/2020/october/columns/nutraceuticals-plant-based-protein-market-grows-stronger.

Muir JG, Rose R, Rosella O, Liels K, Barrett JS, Shepherd SJ, et al. Measurement of Short-Chain Carbohydrates in Common Australian Vegetables and Fruits by High-Performance Liquid Chromatography (HPLC). J Agric Food Chem. 2009 Jan 28;57(2):554–65. Available from: https://doi.org/10.1021/jf802700e.

Murphy K, Garland-Campbell K, Lyon S, Jones S. Evidence of varietal adaptation to organic farming systems. F Crop Res. 2007 Jun 20;102:172–177.

Olesen JE, Askegaard M, Rasmussen IA. Design of an Organic Farming Crop-Rotation Experiment. Acta Agric Scand Sect B — Soil Plant Sci [Internet]. 2000 Jan 1;50(1):13–21. Available from: https://doi.org/10.1080/090647100750014367.

Peoples MB, Herridge DF, Ladha JK. Biological nitrogen fixation: An efficient source of nitrogen for sustainable agricultural production? In: Ladha JK, Peoples MB, editors. Management of Biological Nitrogen Fixation for the Development of More Productive and Sustainable Agricultural Systems: Extended versions of papers presented at the Symposium on Biological Nitrogen Fixation for Sustainable Agriculture at the 15th Congress [Internet]. Dordrecht: Springer Netherlands; 1995. p. 3–28. Available from: https://doi.org/10.1007/978-94-011-0055-7_1.

Powers S, Boatwright JL, Thavarajah D. Genome-wide association studies of mineral and phytic acid concentrations in pea (Pisum sativum L.) to evaluate biofortification potential. G3 Genes|Genomes|Genetics. 2021 Jul 7; Available from: https://doi.org/10.1093/g3journal/jkab227.

Powers S, Mirsky E, Bandaranayake A, Thavarajah P, Shipe E, Bridges W, et al. Field pea (Pisum sativum L.) shows genetic variation in phosphorus use efficiency in different P environments. Sci Rep. 2020;10(1):18940. Available from: https://doi.org/10.1038/s41598-020-75804-0.

Powers SE, Thavarajah D. Checking Agriculture's Pulse: Field Pea (Pisum Sativum L.), Sustainability, and Phosphorus Use Efficiency. Vol. 10, Frontiers in Plant Science . 2019. p. 1489. Available from: https://www.frontiersin.org/article/10.3389/fpls.2019.01489.

Ray H, Bett K, Tar'an B, Vandenberg A, Thavarajah D, Warkentin T. Mineral micronutrient content of cultivars of field pea, chickpea, common bean, and lentil grown in Saskatchewan, Canada. Crop Sci. 2014;54(4).

Reganold JP, Wachter JM. Organic agriculture in the twenty-first century. Nat Plants [Internet]. 2016;2(2):15221. Available from: https://doi.org/10.1038/nplants.2015.221.

SAS Institute Inc. User's guide: Statistics SAS Institute, Version 9.4. Cary, NC, 2013.

Shirtliffe SJ, Johnson EN. Progress towards no-till organic weed control in western Canada. Renew Agric Food Syst. 2012;27(1):60–67.

Snapp SS, Swinton SM, Labarta R, Mutch D, Black JR, Leep R, et al. Evaluating Cover Crops for Benefits, Costs and Performance within Cropping System Niches. Agron J. 2005;97:322–32. Available from: http://dx.doi.org/10.2134/agronj2005.0322.

Thavarajah D, Abare A, Mapa I, Coyne C, Thavarajah P, Kumar S. Selecting Lentil Accessions for Global Selenium Biofortification. Plants. 2017 Aug 26;6(3):34. Available from: http://www.mdpi.com/2223-7747/6/3/34.

Thavarajah D, Siva N, Johnson N, McGee R, Thavarajah P. Effect of cover crops on the yield and nutrient concentration of organic kale (Brassica oleracea L. var. acephala). Sci Rep. 2019;9(1).

Thavarajah, D., Johnson CR, McGee R, Thavarajah P. Phenotyping nutritional and antinutritional traits. In: Phenomics in Crop Plants: Trends, Options and Limitations. 2015.

Trewavas A. Urban myths of organic farming. Nature [Internet]. 2001;410(6827):409–10. Available from: https://doi.org/10.1038/35068639.

USAPulses. Processing Information and Technical Manual [Internet]. Available from: https://www.usapulses.org/technical-manual/chapter-3-production/production.

USDA. USDA Organic Agriculture Program [Internet]. 2016. Available from: https://nifa.usda.gov/program/organic-agriculture-program.

Vandemark G, Thavarajah S, Siva N, Thavarajah D. Genotype and Environment Effects on Prebiotic Carbohydrate Concentrations in Kabuli Chickpea Cultivars and Breeding Lines Grown in the U.S. Pacific Northwest. Front Plant Sci [Internet]. 2020;11:112. Available from: https://www.frontiersin.org/article/10.3389/fpls.2020.00112.

Wang N, Hatcher DW, Toews R, Gawalko EJ. Influence of cooking and dehulling on nutritional composition of several varieties of lentils (Lens culinaris). LWT - Food Sci Technol [Internet].

2009;42(4):842–8. Available from: https://www.sciencedirect.com/science/article/pii/S0023643808002600.

Wang S, Sharp P, Copeland L. Structural and functional properties of starches from field peas. Food Chem [Internet]. 2011;126(4):1546–52. Available from: https://www.sciencedirect.com/science/article/pii/S0308814610015827.

Wiebe L, Fox SL, Entz MH. Organic selection may improve yield efficiency in spring wheat: a preliminary analysis. Can J Plant Sci [Internet]. 2016 Oct 28;97(2):298–307. Available from: https://doi.org/10.1139/cjps-2016-0141.

Table 1: Experimental design used in the dry pea nutritional breeding trials.

Year (location)	2019 (Clemson; Pelion), 2020 (Pelion)
Location	Clemson, SC; Pelion SC
Replicates (Year)	2 (2019); 3(2020)
Cultivars/ Breeding lines	**Cultivars (25)**: AAC Carver, AAC Comfort, AC Agassiz, AC Earlystar, Banjo, CDC Amarillo, CDC Gwater, CDC Inca, CDC Saffron, CDC Spectrum, CDC Striker, Delta, DS Admiral, Durwood, Fiddle Flute, Hampton, Jetset, Korando, LG Koda, Matrix, Mystique, Nette 2010, SW Arcadia, SW Midas **Breeding lines (19)**: PS01100925, PS03101445, PS05100735, PS08100582, PS08101004, PS08101022, PS12100047, PS14100079, PS1410B0003, PS1410B0006, PS1410B0065, PS1410B0073, PS1514B0002, PS16100003, PS16100038, PS16100085, PS16100086, PS16100096, PS16100127
Total	308

Table 2: Mean monthly temperature and precipitation for two growing locations in SC, USA.

Year	Location	Source	Jan	Feb	Mar	Apr	May
2019	Clemson	Temp (°C)	6.1	10.0	10.8	16.9	23.1
		Precipitation (mm)	140	193	88.9	117	19.3
	Pelion	Temp (°C)	9.4	12.8	13.6	19.4	25.6
		Precipitation (in)	3.6	1.7	2.6	4.3	2.7
2020	Pelion	Temp (°C)	9.6	11.0	16.6	17.6	20.8
		Precipitation (in)	69	172	83	81	236

Table 3: Soil chemical properties at the locations where dry pea was grown in 2019 and 2020.

Year	Location (Soil type)	Soil pH	N-NO_3 (PPM)	P (lbs/ac)	K (lbs/ac)	Organic Matter (%)
2019	Clemson (Clay loam)	6.3	48	76	284	4.3
	Pelion (Sandy)	6.8	16	727	108	0.8
2020	Pelion (Sandy)	7.1	21	549	81	1.1

Table 4: Analysis of variance and broad-sense heritability estimates of yield and nutritional traits evaluated for dry pea genotypes tested in SC, USA.

Component	Cultivar	Location	Year	Cultivar × Location	Cultivar × Year	H^2
**Yield**	**	*	**	**	*	0.21
**TSW**	**	**	**	**	**	0.69
**Protein**	**	**	**	NS	**	0.24
Prebiotic carbohydrates						
Sugar Alcohols						
Myo-Inositol	**	**	**	**	**	0.52
Xylitol	**	NS	**	**	**	0.66
Galactinol	**	**	**	**	**	0.74
Sorbitol	**	**	NS	**	NS	0.42
Mannitol	**	NS	**	NS	**	0.57
Simple Sugars						
Glucose	**	**	NS	NS	NS	0.29
Fructose	**	**	**	NS	**	0.27
Sucrose	**	**	**	**	NS	0.52
Arabinose	**	NS	**	**	**	0.65
Maltose	NS	**	**	NS	NS	0.00
RFO and FOS						
Sta+Raf	**	**	**	*	**	0.64
Ver+Kes	**	NS	**	**	**	0.75
Nystose	**	**	**	NS	*	0.27
Starch Polysaccharides						
Resistant starch	NS	**	*	NS	**	0.00
Total starch	NS	**	**	NS	NS	0.00
**Minerals**						
K	**	**	**	*	NS	0.07
Ca	*	**	**	NS	NS	0.03
Mg	*	**	NS	NS	NS	0.00
P	**	NS	NS	NS	NS	0.02

Fe	**	**	**	**	NS	0.00
Zn	**	**	**	NS	NS	0.03
Mn	*	NS	NS	NS	NS	0.00
Cu	**	NS	NS	NS	NS	0.00
Se	NS	**	**	NS	NS	0.00
Phytic acid	*	NS	**	NS	NS	0.00

Raffinose family of oligosaccharides (RFO); Fructooligosaccharides (FOS); Stachyose, and Raffinose (Sta+Raf); Verbascose and Kestose (Ver+Kes); ** significant at $P<0.05$; * significant at $P<0.1$; Not significant (NS); H^2 broad-sense heritability estimate.

Table 5: Range and mean nutrient concentrations of organic dry pea grown in SC.

Nutrient	Organic		%RDA	
	Range	Mean	Female	Male
**Protein (g/100 g)**	12.6-34.2	21.1	27-74(46)	23-61(38)
Prebiotic carbohydrates				
Sugar Alcohols (mg/100 g)				
Myo-Inositol	98-399	244		
Xylitol	2.5-31.7	15.7		
Galactinol	91.3-425	163		
Sorbitol	8.4-115	34.9		
Mannitol	0.9-23.8	5.9		
Simple Sugars (mg/100 g)				
Glucose	14.6-137	62		
Fructose	1.7-30.7	6.4		
Sucrose	1530-3043	2156		
Arabinose	3.3-13.1	7.2		
Maltose	2.1-289	26.3		
RFO and FOS (mg/100 g)				
Sta+Raf	2111-4077	3128		
Ver+Kes	1548-3929	2688		

Nystose	1.6-9.1	3.4		
Starch Polysaccharides (g/100 g)				
Resistant starch	4.2-10	7.6		
Total starch	35.4-66.9	52.6		
Total known prebiotic carbohydrates *(g/100 g)*	12.5-19.8	16.1	63-99 (81)	63-99 (81)
Minerals (mg/100 g)				
Potassium (K)	322-1716	1008	38.8	29.6
Calcium (Ca)	11-338	94	7.8-9.4	9.4
Magnesium (Mg)	46-232	125	39.1- 40.3	31.3
Phosphorus (P)	123-759	377	53.9	53.9
Iron (Fe)	1.9-26.2	5.7	31.7-71.3	71.3
Zinc (Zn)	1.1-7.5	3.2	40.0	29.1
Manganese (Mn)	0.4-3.4	1.2	66.7	52.2
Copper (Cu)	0.2-3.5	0.8	88.9	88.9
Selenium (Se: µg/100 g)	0-130	20	36.4	36.4
Phytic acid (mg/100 g)	88.8-354	159		

Values are based on the combined statistical analysis of 308 data points for the current study (dry weight basis). Total prebiotic carbohydrates include sugar alcohols, simple sugars, raffinose-family oligosaccharides, and resistant starch. % RDA is based on 20 g/day for total prebiotic carbohydrates (22). %RDA for protein is 46 g/day for women aged 19-70+ years and 56 g/day for men aged 19-70+years. Mineral %RDA values are from the National Institute of Health (https://www.ncbi.nlm.nih.gov/books/NBK545442/table/appJ_tab3/?report=objectonly)

Table 6: Correlation of yield, prebiotic carbohydrates, and protein content of organic dry pea genotypes.

Variable	Yield	Myo	Xyl	Gal	Sor	Man	Glu	Fru	Suc	Ara	Mal	Sta+Raf	Ver+Kes	Nys	RS	SS	TS	Pro
Yield	-																	
Myo-Inositol (Myo)	NS	-																
Xylitol (Xyl)	-**	NS	-															
Galactinol (Gal)	**	**	-**	-														
Sorbitol (Sor)	**	**	-**	**	-													
Mannitol (Man)	-**	**	**	-**	NS	-												
Glucose (Glu)	**	**	NS	**	**	NS	-											
Fructose (Fru)	*	NS	NS	NS	**	**	**	-										
Sucrose (Suc)	-**	**	**	NS	NS	NS	**	**	-									
Arabinose (Ara)	-**	NS	**	-**	NS	**	NS	**	**	-								
Maltose (Mal)	-**	**	*	**	**	*	**	**	**	**	-							
Sta+Raf	**	**	NS	**	**	-**	**	**	**	-**	**	-						
Ver+Kes	**	-**	-**	NS	**	NS	NS	**	**	-**	-**	**	-					
Nystose (Nys)	**	-**	-**	**	NS	-**	**	**	-**	NS	NS	**	**	-				
Resistant starch (RS)	-**	-**	**	**	**	**	-**	-**	**	NS	**	**	-**	**	-			
Soluble starch (SS)	**	-**	NS	**	**	**	**	**	NS	NS	NS	-**	**	-**	-**	-		
Total starch (TS)	**	**	**	-**	**	NS	**	NS	NS	NS	NS	**	NS	NS	NS	**	-	
Protein (Pro)	-**	*	*	NS	NS	**	NS	NS	*	**	**	NS	-**	-**	NS	-*	NS	-

Stachyose and Raffinose (Sta+Raf); Verbascose and Kestose (Ver+Kes); ** significant at *P<0.05*; * significant at *P*<0.1; Not significant (NS).

Table 7: Correlation of yield, critical minerals, and phytic acid concentrations of organic dry pea genotypes.

Variable	Yield	K	Mg	Fe	Zn	P	phytic acid
Yield	-						
K	-*	-					
Mg	**	**	-				
Fe	*	**	**	-			
Zn	NS	**	**	**	-		
P	NS	**	**	**	**	-	
Phytic acid	NS	-*	-*	-*	-**	-*	-

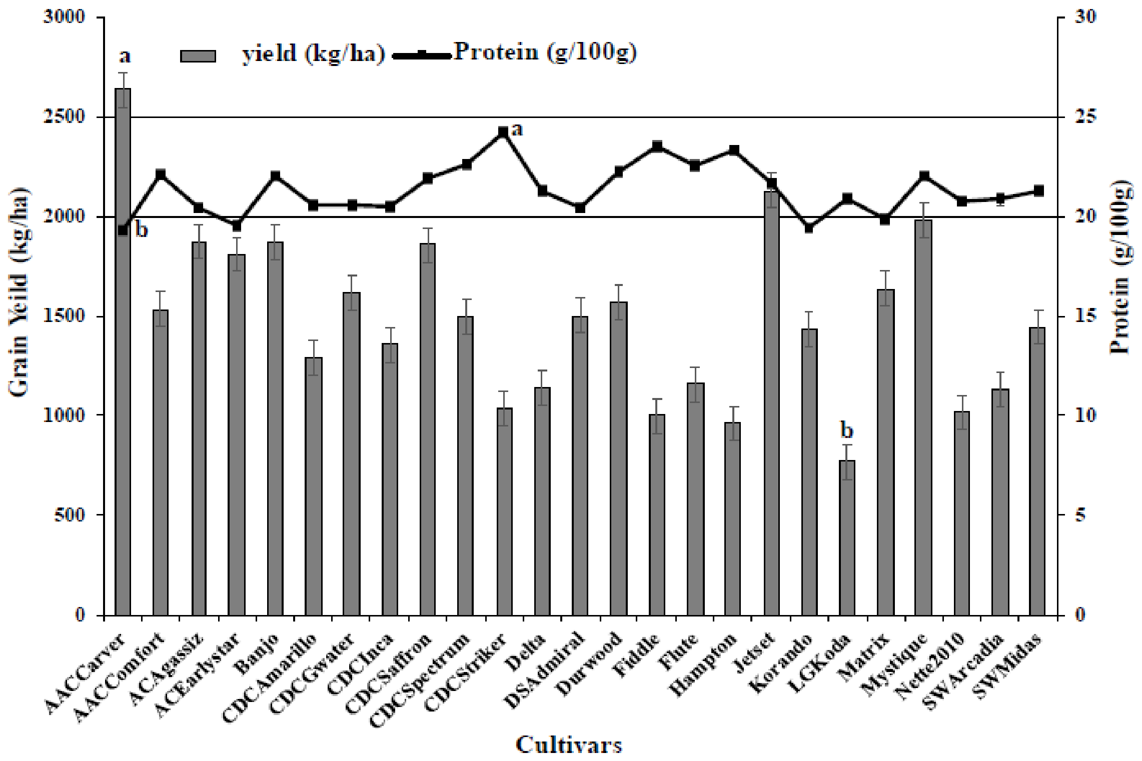

Figure 1: Variation of grain yield and protein content among dry pea cultivars grown in the organic system.

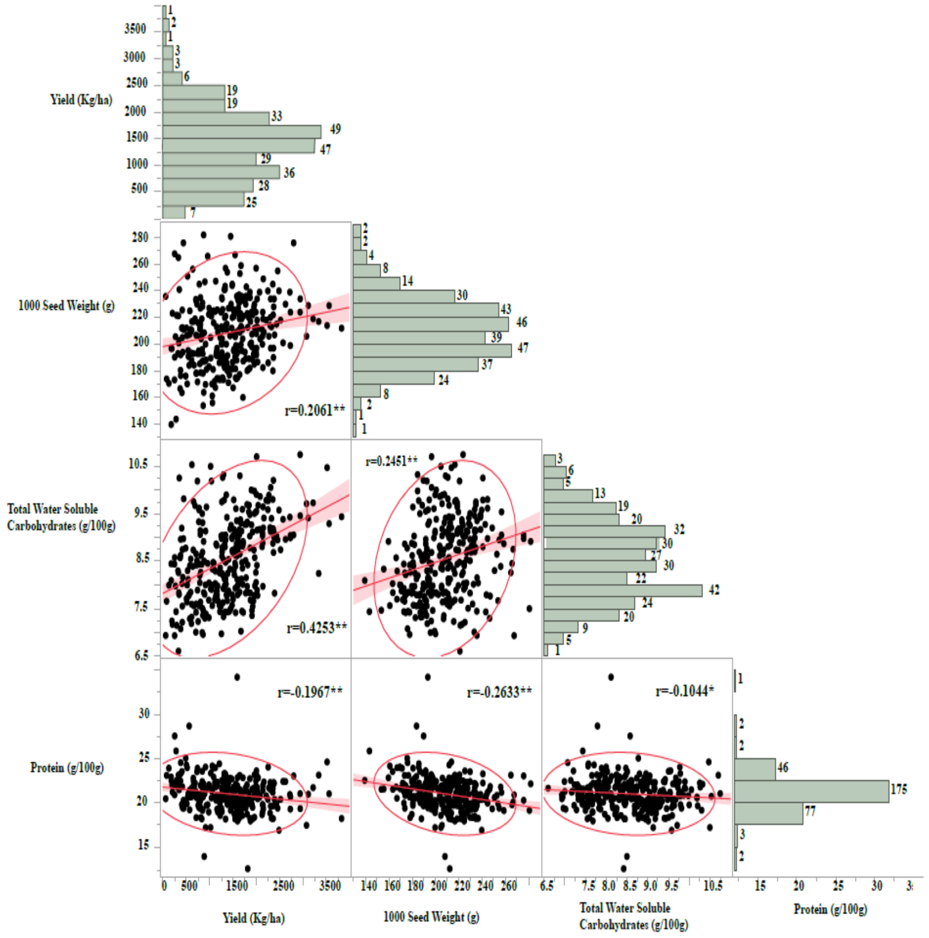

Figure 2: Correlations and distribution of grain yield, 1000 seed weight, total water-soluble carbohydrates, and protein concentration among the genotypes grown under organic field conditions.

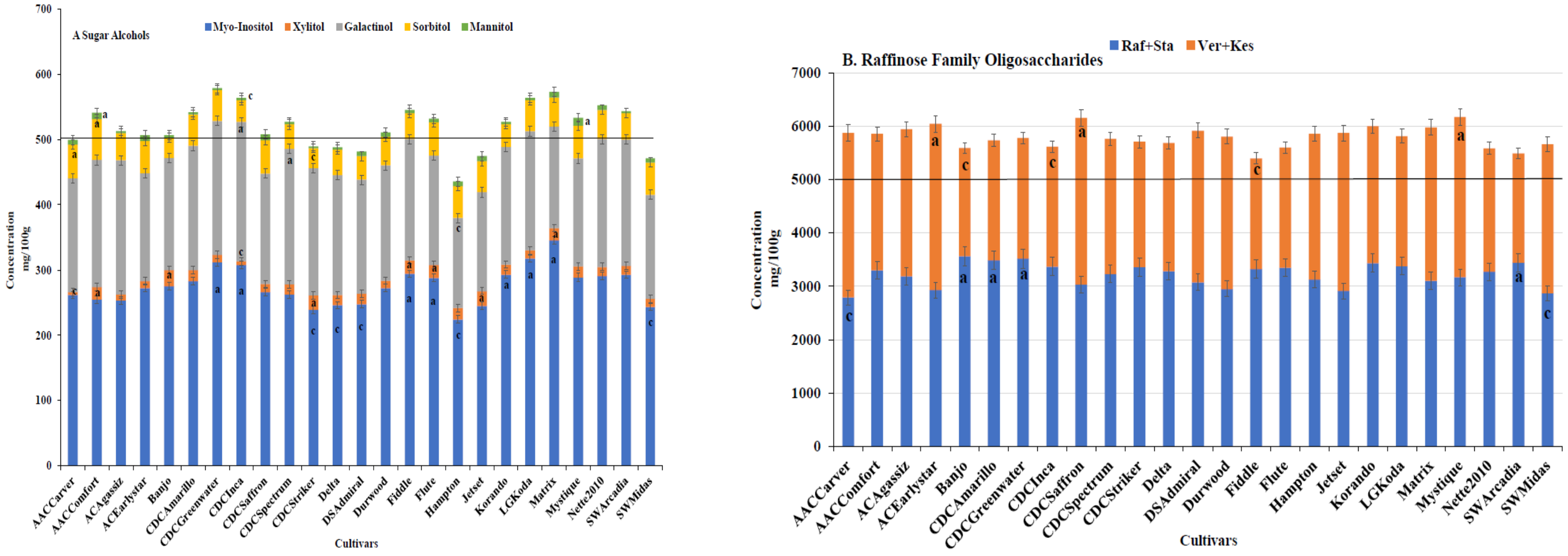

Figure 3: Variation of (A) sugar alcohols and (B) raffinose family oligosaccharides concentrations among dry pea cultivars grown in an organic system.

Click here to access/download
Supporting Information
Suplimentary- data.xlsx
